# Eltroxin and Hesperidin mitigate testicular and renal damage in hypothyroid rats: amelioration of oxidative stress through PPARγ and Nrf2/HO-1 signaling pathway

**DOI:** 10.1186/s42826-024-00204-8

**Published:** 2024-05-14

**Authors:** Hadeel M. Osama, Sally M. Khadrawy, EL-Shaymaa EL-Nahass, Sarah I. Othman, Hanaa M. Mohamed

**Affiliations:** 1 Genetics and Molecular Biology Division, Zoology Department, Faculty of Science, Beni-Suef University, Beni-Suef, Egypt; 2 Pathology Department, Faculty of Veterinary Medicine, Beni-Suef University, Beni-Suef, Egypt; 3 Biology Department, College of Science, Princess Nourah bint Abdulrahman University, P.O. BOX 84428, Riyadh, 11671 Saudi Arabia

**Keywords:** Hesperidin, Hypothyroidism, Testis, Kidney, Nrf2/HO-1, PPARγ, Rat

## Abstract

**Background:**

Thyroid hormones (THs) regulate growth, development and function of different tissues. Hypothyroidism is a common clinical disorder characterized by deficiency in THs and adversely affects the development and functions of several organs. This work aimed to investigate the ameliorative effect of eltroxin (ELT), a hypothyroidism medication, and hesperidin (HSP), a flavonoid, against testicular and renal toxicity in hypothyroid rats. Twenty-four rats were divided into four groups and treated orally for 12 weeks. Group I (control), group II (hypothyroidism) received 20 mg/kg carbimazole (CBZ), group III received CBZ and 0.045 mg/kg ELT, and group IV received CBZ and 200 mg/kg HSP.

**Results:**

CBZ administration induced biochemical and histopathological changes in testis and kidney. Co-administration of ELT or HSP significantly (*P* < 0.05) ameliorated THs, reduced urea and creatinine while raised follicle stimulating hormone (FSH), Luteinizing hormone (LH), and testosterone in serum. Testicular and renal malondialdehyde level as a lipid peroxidation indicator, tumor necrosis factor-α (TNF-α), and interleukin-6 (IL-6) were significantly (*P* < 0.05) decreased while glutathione content, glutathione peroxidase, and glutathione-s-transferase activities were significantly (*P* < 0.05) increased. The histopathological changes were also diminished. Decreased mRNA and protein expressions of nuclear factor erythroid 2–related factor 2 (Nrf2), heme oxygenase-1 (HO-1), and peroxisome proliferator-activated receptor gamma(PPARγ) in hypothyroid rats were up-regulated after ELT or HSP treatment.

**Conclusions:**

ELT and HSP showed antioxidant and anti-inflammatory effects against CBZ-induced testicular and renal toxicity, and these effects may be promoted via activating Nrf2/HO-1 and PPARγ signaling pathways.

## Background

Hypothyroidism (HPO) is a chronic disorder that affects about 10% of the worldwide individuals. It is characterized by a thyroid hormones (THs) insufficient supply of thyroxine (T4) and triiodothyronine (T3), which are important for regulating several metabolic processes, growth and development [1]. Thyroid dysfunction should be properly and promptly treated due to the broad spectrum effects of THs on the body tissues and metabolism [2]. Over time, untreated HPO may result in a number of health problems such as infertility, obesity, joint pain, and heart diseases [3]. According to data from a prior research, low thyroid hormone levels may contribute to an increase in oxidative stress (OS) [4].

THs affect renal morphology and function since they are important for the growth and development. THs deficiency adversely affects the kidney function through altering intra-renal hemodynamics, and renin angiotensin aldosterone system (RAAS), as well as the structural changes including low kidney/body weight ratio, and abnormal glomerular architecture [5]. Similarly, THs regulate some functions in the testis such as sperm motility, non-germ cells′ proliferation and differentiation, steroidogenesis, and testicular antioxidant status. Consequently, declined or raised THs can affect testicular function by different ways [6].

Eltroxin (ELT) (Levothyroxine) is a synthetic form of the thyroid hormone; T4 [7]. Levothyroxine is the cornerstone of HPO therapy and is listed as one of the fundamental healthcare essential medications by the World Health Organization [3]. This synthetic drug elevates serum T3 and T4 levels to regulate the thyroid gland activity [8]. Prolonged use of a synthetic drug can treat HPO, but it may have adverse effects [9]. Therefore, researchers are interested in flavonoids because of their abundance in food, possible health benefits, and relatively low toxicity when compared to other phytochemicals [10]. Hesperidin (HSP), a pharmacologically active flavonoid aglycone, is widely distributed in citrus species (orange, blood orange, lime and lemon) [11]. HSP was reported to have a broad variety of pharmacological effects including anti-thyroid, anti-inflammatory, anti-oxidant, anti-allergic, anti-cancer, anti-viral, anti-microbial, neuroprotective, and cardioprotective properties. It has been shown in several preclinical investigations that it protects against malignant transformation and development by acting on a variety of cellular signaling pathways [12, 13]. The antioxidant activities of HSP include increasing of cellular antioxidant defenses, quenching of free radicals, suppression of reactive oxygen species (ROS) producing enzymes, preventing DNA deterioration [14, 15], and Nrf2 activation [16]. Li et al. [17] has also verified that HSP exhibited a good safety profile when used in animal studies.

Nrf2 is a strategic antioxidant player in the thyroid gland [18]. It has been demonstrated that Nrf2 has a role in the thyroidal function, it affects the basal and the thyroid-stimulating hormone (TSH)-induced boost of thyroglobulin, which is a key protein made by the gland and considers the precursor of T3 and T4 [19]. Also, Nrf2 controls the expression of several anti-oxidant and cytoprotective genes in addition to tissue-specific genes required for the unique functional and homeostatic systems of each specific tissue in different organs [20].

Peroxisome proliferator-activated receptors (PPARs), ligand-inducible transcription factors and members of the nuclear receptor family, have a crucial role in maintaining the cells’ regular activity. When PPARs are turned on, they heterodimerize with the retinoid X receptor (RXR), which in turn regulates the expression of several genes [21]. Three PPAR isoforms have been identified; PPARα, PPARβ/δ, and PPARγ [22]. PPARγ has an important role in male reproduction through regulating the genes for fatty acid metabolism [23]. Also, PPARγ affects kidney development, and lipid metabolism [24]. According to several reports, PPARγ and Nrf2 coactivation was proved to protect against inflammation and OS [25, 26].

Consequently, we aimed to assess the protective effects of ELT and HSP against alterations in kidneys and testes provoked by CBZ-induced HPO in adult male rats, and to suggest the probable mechanism of action through testing the ability to modulate OS and inflammation possibly by activating PPARγ and Nrf2.

## Methods

### Chemicals

CBZ was purchased as tablets from Chemical Industries Development, Giza, Egypt. HSP was shipped from Sigma-Aldrich (St. Louis, MO, USA), and was stored away from sunlight. ELT was purchased from Glaxo Smith Kline GmbH, Germany. Other chemicals were of analytical grade and were obtained from commercial suppliers.

### Experimental animals and treatments

In the current study, twenty-four adult male albino rats (Rattus norvegicus) weighing 130–150 g were purchased from the unit of animal housing in Nahda University (Egypt). The animals were kept in suitable well aerated cages at normal 12 h light/dark cycles, normal humidity (50–60%) and temperature (25 ± 3 °C) with free access to standard food pellets of known composition and water. Before the onset of the experiment, rats were observed for about 14 days to eliminate any possible infection. All animal treatments were accepted by the Institutional Research Ethics Committee of Beni-Suef University, Egypt (BSU-IACUC, No. 020–123). After the period of acclimatization, the rats were allocated into four equivalent groups with each group comprising six rats (*N* = 6), and were treated for 12 weeks.

Group I (CON): Orally gavaged with distilled water for one week, then 1% carboxymethyl cellulose (CMC) for 11 weeks.

All other rats received 20 mg/kg CBZ, dissolved in distilled water, through gastric intubation once/day for one week to induce HPO [27], then were separated into 3 groups and continued for 11 weeks as follows:

Group II (CBZ): Rats received CBZ only throughout the experimental period.

Group III (CBZ + ELT): In addition to CBZ, rats were treated orally with 0.045 mg/kg/day ELT dissolved in distilled water [28].

Group IV (CBZ + HSP): In addition to CBZ, rats were treated orally with 200 mg/kg/day HSP dissolved in 1% CMC [29].

### Blood collection and tissue sampling

Twenty four hours following the determined experimental period, the animals from all groups were anesthetized intraperitoneally by 80 mg/kg ketamine/10 mg/kg xylazine [30], and blood samples were collected from the medial canthus then rats were sacrificed by cervical dislocation. A median abdominal incision was achieved to expose the kidneys and testes which were carefully excised and washed with cold saline.

Serum was separated from blood samples after complete clotting, at room temperature, and centrifugation at 3000 rpm for 15 min. Serum was used for the analysis of testis and kidney function biomarkers and THs.

Samples from the testes and kidneys were homogenized (10% w/v) in cold phosphate buffered saline, centrifuged at 3000 rpm for 10 min, then the clear supernatant was separated and stored at -20 °C for the analysis of lipid peroxidation, antioxidants, TNF-α and IL-6. Other testis and kidney samples were fixed in Bouin’s solution and 10% neutral buffered formalin, respectively, for the histological processing. The remaining samples were kept at -80 °C for RNA isolation and western blotting.

### Biochemical assays

#### Determination of serum thyroid hormones concentration

Total thyroxine (TT4), total triiodothyronine (TT3) and TSH concentrations of all groups were determined in the serum using ELISA kits acquired from Cal biotech INC (CBI, USA Company) according to the manufacturers’ instructions and following the method of Thakur et al. [31], Maes et al. [32], and Burger and Patel [33] respectively. This assay uses the competitive inhibition enzyme immunoassay technique and the optical density was measured spectrophotometrically at 450 nm using Hitachi spectrophotometry (Tokyo, Japan).

#### Determination of serum testis hormones concentration

Luteinizing hormone (LH), follicle stimulating hormone (FSH), and testosterone levels were measured in the blood serum by enzyme immunoassay kits (ALPCO Diagnostics) following the manufacturer’s instructions.

#### Determination of kidney function biomarkers

Serum urea and creatinine were assayed by Spinreact commercial kits (Spain) depending on the methods of Coulombe and Favreau [34] and Larsen [35] respectively.

#### Determination of pro-inflammatory cytokines

TNF-α and IL-6 were estimated in testicular and renal tissue homogenates using R&D systems ELISA kits (USA) following the manufacturer’s guidelines.

#### Determination of oxidative stress and antioxidant markers

In the homogenate of the testis and kidney, lipid peroxidation was assayed as malondialdehyde (MDA) level according to Preuss et al. [36]. Also, reduced glutathione (GSH), glutathione peroxidase (GPx) and glutathione-s-transferase (GST) were determined following the methods of Beutler et al. [37], Matkovics et al. [38] and Mannervik and Guthenberg [39] respectively.

### Histological examination

Twenty-four hours fixed renal and testicular tissues were washed with distilled water, dehydrated in an ascending ethanol series and paraffin wax embedded. For light microscopy inspection, paraffin sections were cut at a thickness of 4–5 μm via a rotary microtome and were stained with hematoxylin and eosin (H&E) [40]. In each group, six sections were examined. At least eight microscopic fields per each section were scanned. Histopathological lesions of testis and kidney were examined and evaluated with a score formerly described as follows: (–) no influence, (+) mild (< 25% of all fields showed alterations), (++) moderate (< 50% of all fields showed alterations) or (+++) severe changes (< 75% of all fields showed alterations) [41].

### Gene expression analysis by qRT-PCR

Renal and testicular samples were treated with a TRIzol Plus RNA Purification Kit. The purity and concentration of RNA were detected spectrophotometrically. RNAs with A 260/280 ≥ 1.8 were chosen for cDNA synthesis. Up to 2 µg RNA was converted to cDNA by High-Capacity cDNA Reverse Transcription Kit. cDNA was amplified by Fast SYBR™ Green Master Mix using a set of two PCR primers that flank the target region as shown in Table 1. All kits were purchased from ThermoFisher Scientific, USA. RT-PCR conditions were set to 95 °C for 3 min, followed by 35 cycles involving denaturation (95 °C for 15 s), annealing (60 °C for 30 s) and extension (72 °C for 30 s). After PCR run, obtained data were evaluated by 2^-ΔΔCT^ method [42]. The results were standardized to β-actin.

**Table 1:** Primer sequences used for qRT-PCR

Gene symbol	Primer sequence from 5′- 3′	GenBank accession number	Gene Size (bp)
**β-actin**	F: TCCGTCGCCGGTCCACACCC R: TCACCAACTGGGACGATATG	NM_031144.3	1293
**Nrf2**	F: AGCAGGACATGGATTTGATT R: CTTCTCCTGTTCCTTCTGGA	XM_032903520.1	2305
**HO-1**	F: CAGTCGCCTCCAGAGTTTCC R: GTACAAGGAGGCCATCACCAGA	XM_032887931.1	870
**PPARγ**	F: GACCTGAAGCTCCAAGAATACCA R: GCTGGGTCTTTTCAGAATAATAAGG	XM_032905881.1	1515

### Protein expression analysis by Western blotting

Frozen specimens at -80 °C were homogenized in an appropriate lysis buffer (RIPA) containing proteinase inhibitors, then centrifuged for 10 min to assay the protein concentration in the clear supernatant by Bradford reagent (ThermoFisher Scientific, USA). Proteins were separated by sodium dodecyl sulfate-polyacrylamide gel electrophoresis (SDS-PAGE), transferred to PVDF membranes, and blocked with a blocking buffer (5% skimmed milk dissolved in TBS/Tween 20 (TBST)). The membranes were incubated with primary antibodies, against Nrf2, HO-1, PPARγ, and β-actin (Santa Cruz Biotechnology, USA) diluted in the blocking buffer. After washing with TBST, the membranes were probed with secondary antibodies then washed. The bands were visualized with an enhanced chemiluminescence reagent (Bio-Rad, USA), quantified using Image J and normalized to β-actin.

### Statistical analysis

Data were analyzed by SPSS (v.20). Results were represented as mean ± standard deviation (SD), and one-way ANOVA test was used to compare all data statistically, followed by Tukey’s test post hoc analysis. A *P* value < 0.05 was stated significant.

## Results

### Effect of ELT and HSP on circulating THs in CBZ-induced rats

The data recorded in Table 2 showed that CBZ-induced rats had a significant (*P* < 0.001) decrease in serum levels of TT3 and TT4 and a significant (*P* < 0.001) increase in serum level of TSH when compared with the control group. Treatment of hypothyroid rats with ELT or HSP showed significantly (*P* < 0.001) ameliorated serum levels of TT3, TT4, and TSH compared to CBZ-treated group.

**Table 2:** Effect of ELT and HSP on serum TT3, TT4, and TSH hormones levels of hypothyroid rats

Parameter	CON	CBZ	CBZ + ELT	CBZ + HSP
**Total T3 (ng/dl)**	90.75 ± 4.13	54.62 ± 5.52^***^	71.5 ± 5.52^***###^	73.5 ± 5.55^***###^
**Total T4 (ug/dl)**	6.00 ± 0.22	2.21 ± 0.40^***^	4.08 ± 0.75^***###^	4.26 ± 0.97^***###^
**TSH (uIU/ml)**	1.30 ± 0.22	4.29 ± 0.96^***^	2.52 ± 0.58^**###^	2.27 ± 0.58^*###^

### Effect of ELT and HSP on FSH, LH, and testosterone hormones in CBZ-induced rats

The concentration levels of FSH, LH, and testosterone were significantly (*P* < 0.001) decreased in hypothyroid rats when compared with the control group. Treatment of hypothyroid rats with ELT significantly increased FSH (*P* < 0.01), LH (*P* < 0.001), and testosterone (*P* < 0.001) levels. Similarly, treatment of hypothyroid rats with HSP significantly (*P* < 0.001) increased FSH, LH, and testosterone levels as illustrated in Table 3.

**Table 3:** Effect of ELT and HSP on serum FSH, LH, and testosterone hormones levels of hypothyroid rats

Parameter	CON	CBZ	CBZ + ELT	CBZ + HSP
**FSH (mIU/ml)**	2.92 ± 0.45	0.87 ± 0.18^***^	1.49 ± 0.33^***##^	1.67 ± 0.37^***###^
**LH (mIU/ml)**	3.69 ± 0.61	0.90 ± 0.15^***^	1.99 ± 0.29^***###^	1.93 ± 0.37^***###^
**Testosterone (ng/ml)**	3.65 ± 0.40	1.04 ± 0.18^***^	1.99 ± 0.45^***###^	2.28 ± 0.46^***###^

### Effect of ELT and HSP on renal function biomarkers in CBZ-induced rats

As shown in Table 4, the levels of serum urea and creatinine were significantly elevated (*p* < 0.001) in CBZ-induced group compared with the control group. Co-administration of ELT or HSP with CBZ significantly (*p* < 0.001) lowered serum levels of urea and creatinine comparing with the hypothyroid group.

**Table 4:** Effect of ELT and HSP on serum urea and creatinine of hypothyroid rats

Parameter	CON	CBZ	CBZ + ELT	CBZ + HSP
**Urea (mg/dl)**	19.75 ± 3.45	49.12 ± 4.25^***^	34.62 ± 4.06^***###^	31.37 ± 3.50^***###^
**Creatinine (mg/dl)**	1.19 ± 0.18	2.73 ± 0.32^***^	2.06 ± 0.27^***###^	2.10 ± 0.26^***###^

### Effect of ELT and HSP on pro-inflammatory cytokine production in testis of CBZ-induced rats

**Fig. 1:**
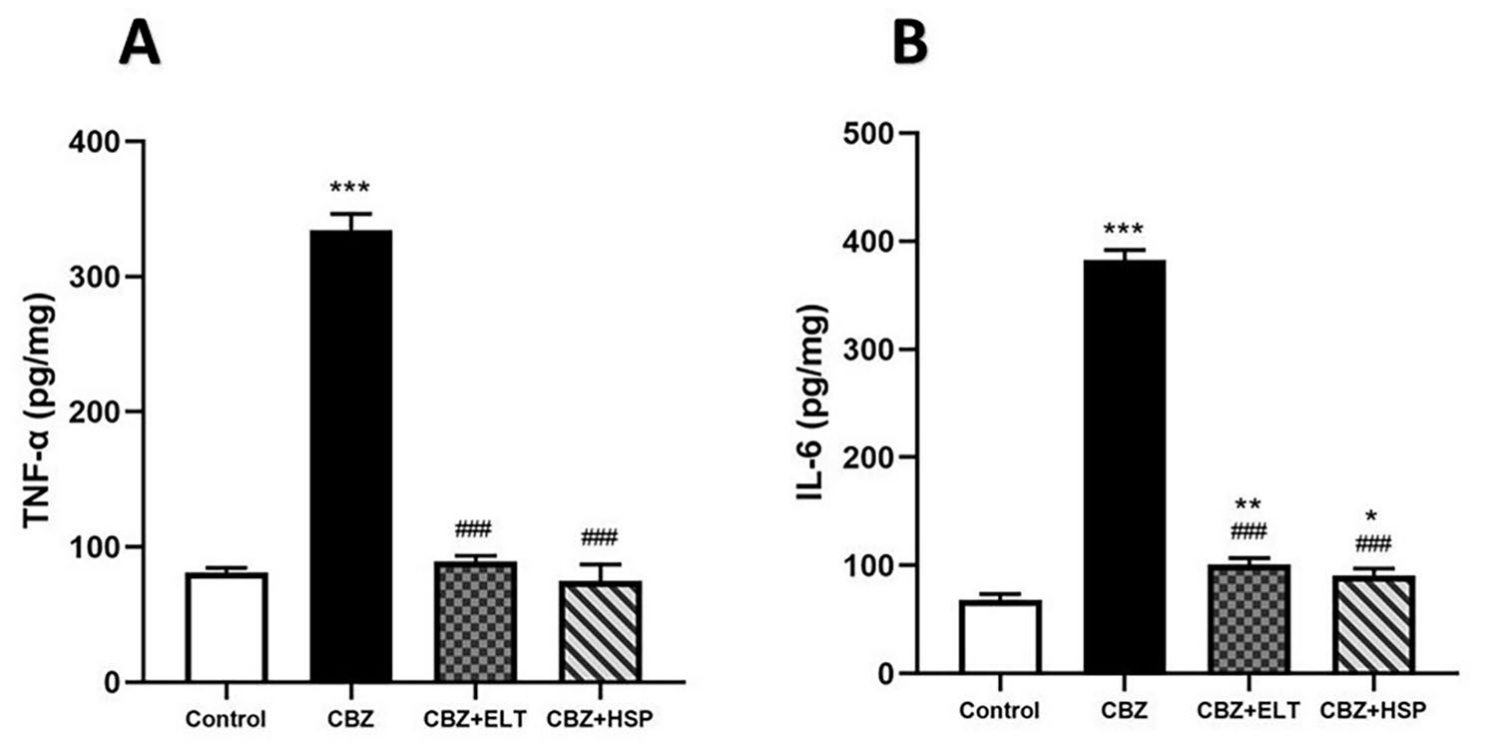
Effect of eltroxin and hesperidin on pro-inflammatory cytokine production in testis of CBZ-induced rats

To investigate the protection by ELT and HSP against HPO-associated inflammation, tissue levels of the pro-inflammatory cytokines (TNF-α and IL-6) were determined. The levels of TNF-α (Fig. 1A) and IL-6 (Fig. 1B) in hypothyroid rats showed a significant (*P* < 0.001) increase when compared with the corresponding control group. Oral supplementation of either ELT or HSP with CBZ produced a significant (*P* < 0.001) decrease in the pro-inflammatory cytokines as shown in Fig. 1.

### Effect of ELT and HSP on pro-inflammatory cytokine production in kidney of CBZ-induced rats

**Fig. 2:**
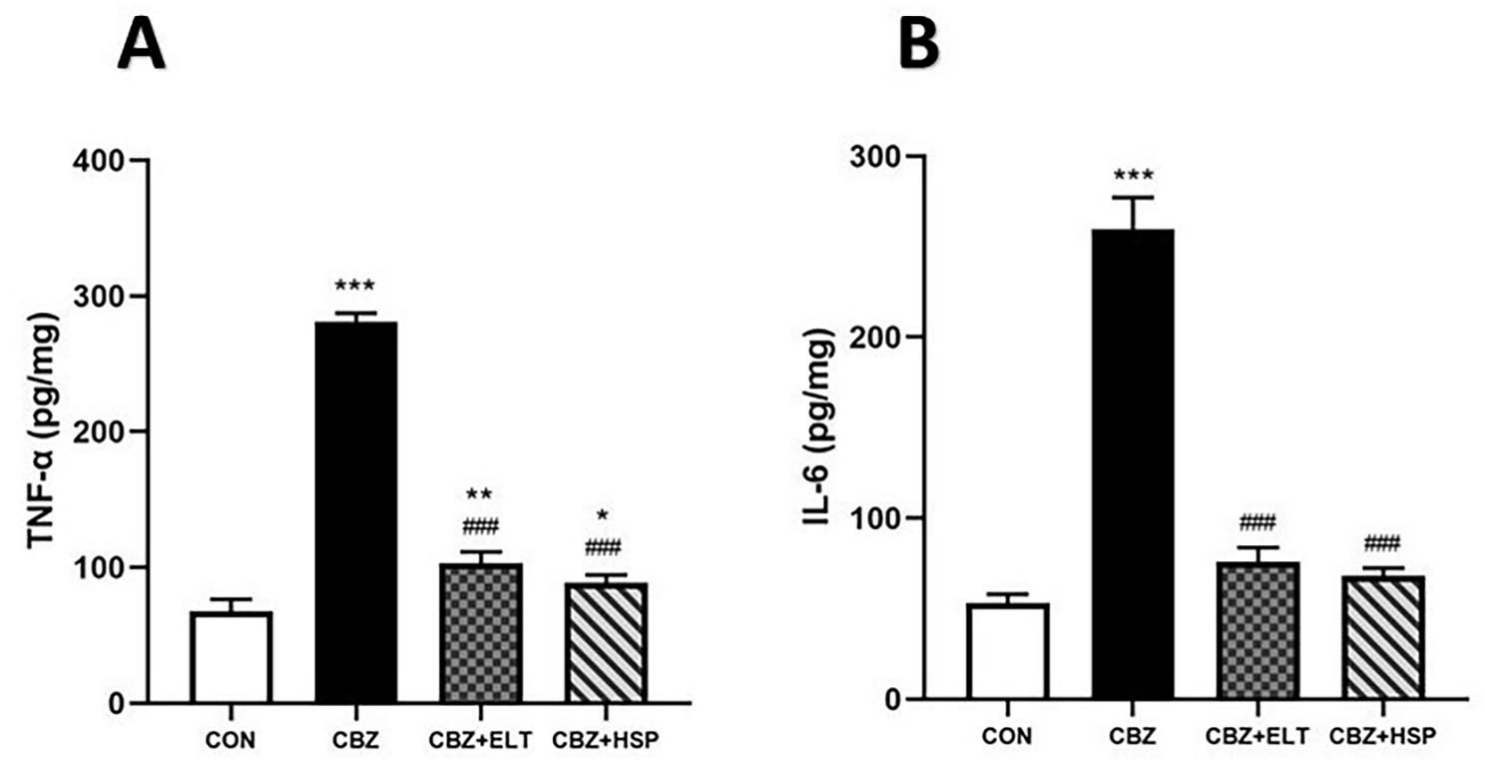
Effect of eltroxin and hesperidin on pro-inflammatory cytokine production in kidney of CBZ-induced rats

TNF-α (Fig. 2A) and IL-6 (Fig. 2B) were significantly (*P* < 0.001) increased in the renal tissues of hypothyroid rats compared to the control rats. Treatment of CBZ-induced rats with ELT or HSP markedly (*P* < 0.001) decreased both TNF-α and IL-6 comparing with the hypothyroid rats as shown in Fig. 2.

### Effect of ELT and HSP on testicular oxidant/antioxidant status in CBZ-induced rats

Hypothyroid rats showed a significant (*P* < 0.001) elevation in lipid peroxidation level when compared with the controls (Table 5). Treating hypothyroid rats with ELT or HSP significantly (*P* < 0.001) reduced lipid peroxidation level. On the contrary, hypothyroid rats showed a marked (*P* < 0.001) decrease in testicular GSH content, GST and GPX activities when compared with the control rats. Co-administration of ELT or HSP to hypothyroid rats significantly (*P* < 0.001) increased GSH, GST and GPX compared to CBZ-induced group (Table 5).

**Table 5:** Effect of ELT and HSP on testicular oxidant/antioxidant status of hypothyroid rats

Parameter	CON	CBZ	CBZ + ELT	CBZ + HSP
**MDA (nmol/mg protein)**	0.62 ± 0.10	2.73 ± 0.15^***^	1.22 ± 0.08^***###^	1.07 ± 0.03^**###^
**GSH (µg/mg protein)**	1.73 ± 0.15	0.40 ± 0.10^***^	1.40 ± 0.09^*###^	1.43 ± 0.05^*###^
**GST (µg/mg protein)**	1.96 ± 0.15	0.56 ± 0.15^***^	1.60 ± 0.09^*###^	1.80 ± 0.10^###^
**GPX (nmol/mg protein)**	42.60 ± 4.10	11.23 ± 1.23^***^	32.40 ± 1.25^**###^	36.03 ± 1.50^*###^

### Effect of ELT and HSP on renal oxidant/antioxidant status in CBZ-induced rats

Lipid peroxidation level was elevated significantly (*P* < 0.001) in CBZ-induced rats compared to the control rats. Administration of ELT or HSP with CBZ showed a noticeable (*P* < 0.001) modulation in lipid peroxidation level as shown in Table 6. In contrast, GSH, GST, and GPX were significantly (*P* < 0.001) decreased in CBZ-induced rats compared to the control rats, while co-administration of ELT or HSP with CBZ showed a significant increase in GSH (*p* < 0.01), GST (*p* < 0.001) and GPX (*p* < 0.001) (Table 6).

**Table 6:** Effect of ELT and HSP on renal oxidant/antioxidant status of hypothyroid rats

Parameter	CON	CBZ	CBZ + ELT	CBZ + HSP
**MDA (nmol/mg protein)**	0.70 ± 0.06	2.33 ± 0.15 ^***^	1.11 ± 0.04^*###^	0.95 ± 0.17^###^
**GSH (µg/mg protein)**	1.46 ± 0.15	0.50 ± 0.09^***^	1.06 ± 0.15^*##^	1.10 ± 0.09^*##^
**GST (µg/mg protein)**	1.80 ± 0.09	0.66 ± 0.15^***^	1.26 ± 0.11^**###^	1.43 ± 0.05^*###^
**GPX (nmol/mg protein)**	38.40 ± 4.35	14.46 ± 1.25^***^	29.53 ± 1.84^*###^	29.73 ± 1.59^*###^

### Effect of ELT and HSP on gene and protein expression levels of Nrf2, HO-1 and PPARγ in the testis of CBZ-induced rats

**Fig. 3:**
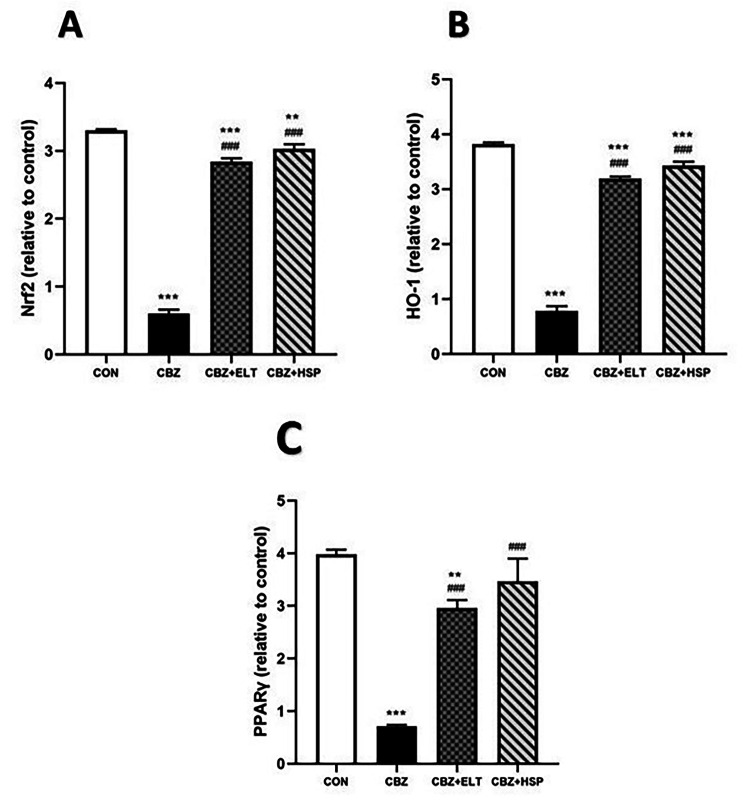
Effect of eltroxin and hesperidin on gene expression level of Nrf2, HO-1 and PPARγ in the testis of CBZ-induced rats

We examined the efficacy of Nrf2/HO-1 signaling and PPARγ in defining the molecular mechanism controlling the protective impact of ELT and HSP on CBZ-induced hypothyroidism in rats. Nrf2 (Fig. 3A), HO-1 (Fig. 3B) and PPARγ (Fig. 3C) mRNA abundance in the testis of CBZ-induced group showed a significant (*P* < 0.001) down-regulation when compared with the corresponding control group. Oral treatment of the hypothyroid rats with either ELT or HSP produced a marked (*P* < 0.001) up-regulation of Nrf2, HO-1 and PPARγ mRNA expression in the testis (Fig. 3).

**Fig. 4:**
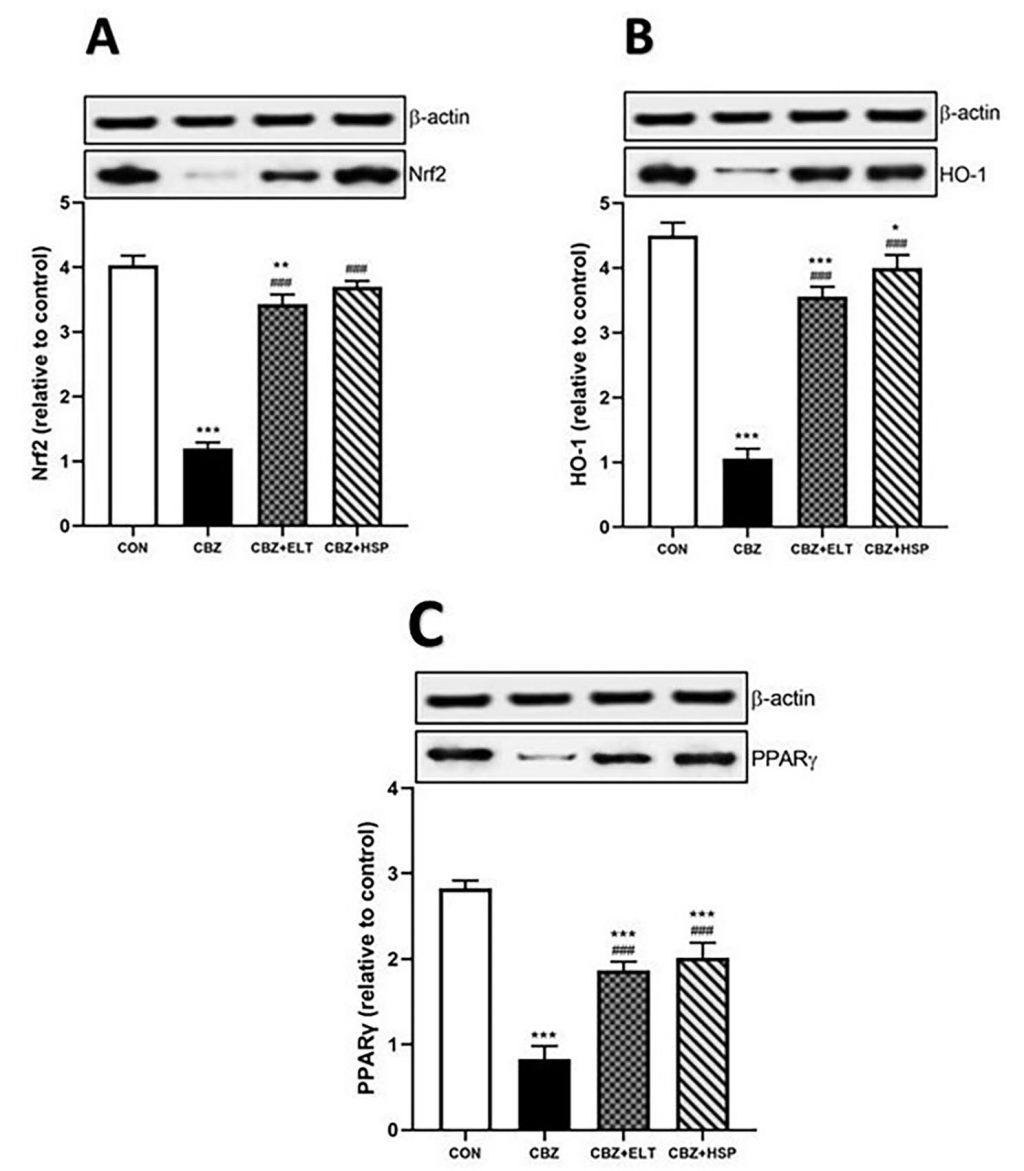
Effect of eltroxin and hesperidin on protein expression level of Nrf2, HO-1 and PPARγ in the testis of CBZ-induced rats

Western blotting data showed a significant (*P* < 0.001) decrease in the protein expression level of Nrf2 (Fig. 4A), HO-1 (Fig. 4B) and PPARγ (Fig. 4C) in the testes of CBZ-induced rats when compared to the control rats. Oral supplementation of either ELT or HSP produced a significant (*P* < 0.001) increase in the protein expression level of Nrf2, HO-1 and PPARγ in the testes of the hypothyroid rats (Fig. 4).

### Effect of ELT and HSP on gene and protein expression levels of Nrf2, HO-1, and PPARγ in the kidney of CBZ-induced rats

**Fig. 5:**
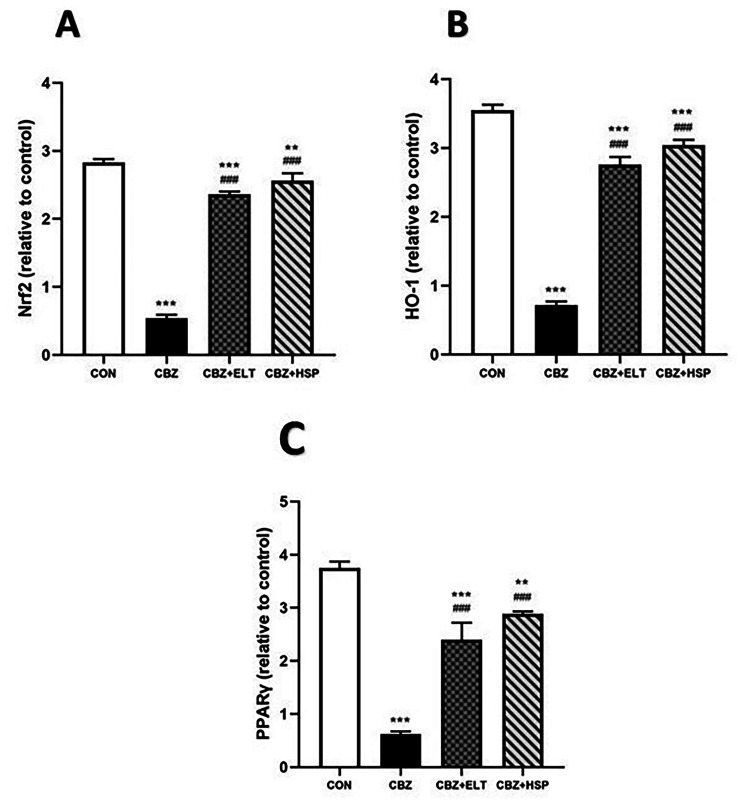
Effect of eltroxin and hesperidin on gene expression level of Nrf2, HO-1, and PPARγ in the kidney of CBZ-induced rats

**Fig. 6:**
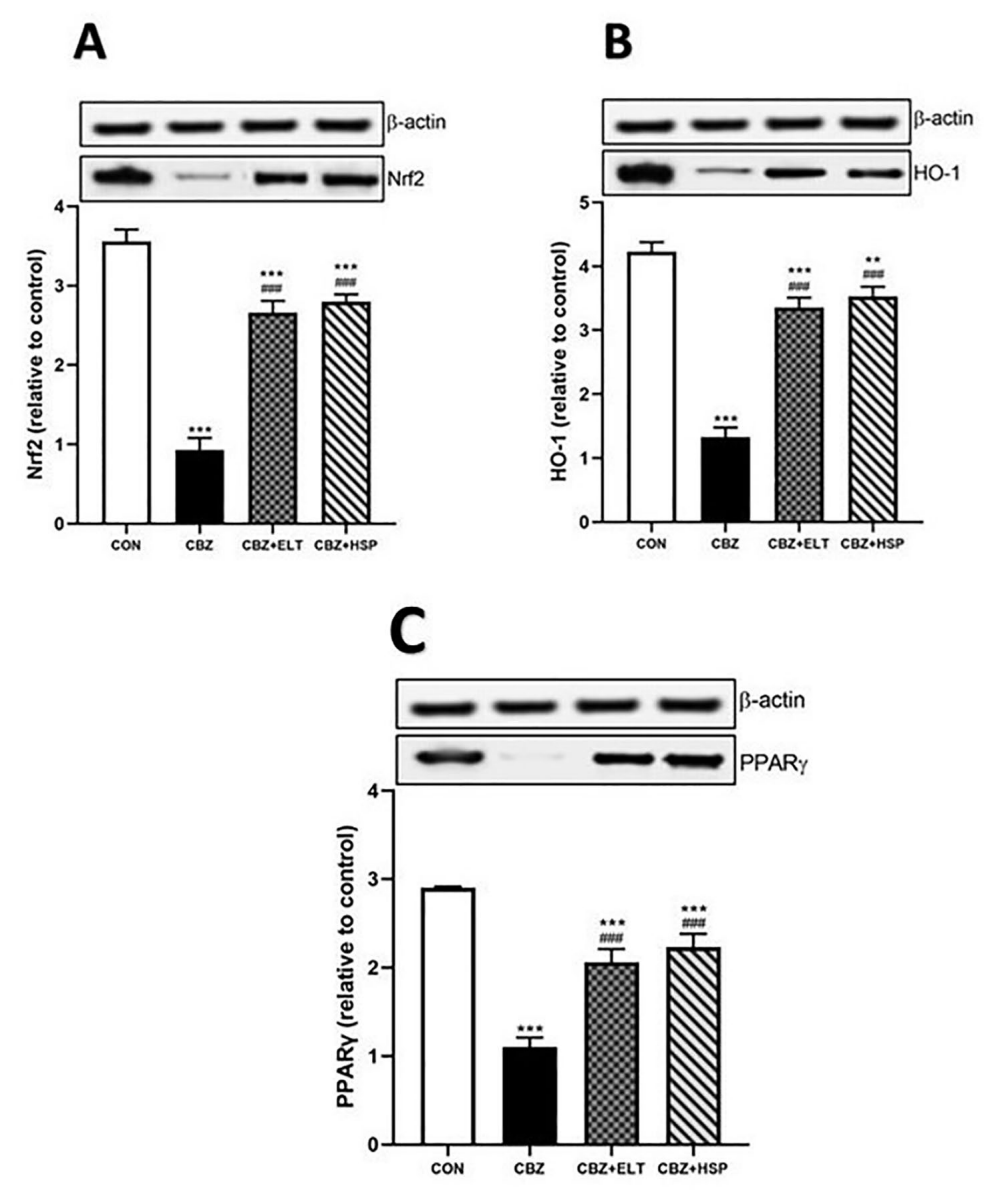
Effect of eltroxin and hesperidin protein expression level of Nrf2, HO-1, and PPARγ in the kidney of CBZ-induced rats

Nrf2 (Fig. 5A), HO-1 (Fig. 5B), and PPARγ (Fig. 5C) mRNA abundance in the kidney of hypothyroid rats showed a significant (*P* < 0.001) decrease when compared with the control rats. Similarly, protein expression levels of Nrf2 (Fig. 6A), HO-1 (Fig. 6B), and PPARγ (Fig. 6C) were significantly (*P* < 0.001) down-regulated in the kidney of hypothyroid rats. Treatment of the hypothyroid rats with ELT or HSP produced a significant (*P* < 0.001) up-regulation of Nrf2, HO-1, and PPARγ mRNA and protein expression levels as shown in Figs. 5 and 6.

### Effect of ELT and HSP on histopathological changes in the testis of CBZ-induced rats

**Fig. 7:**
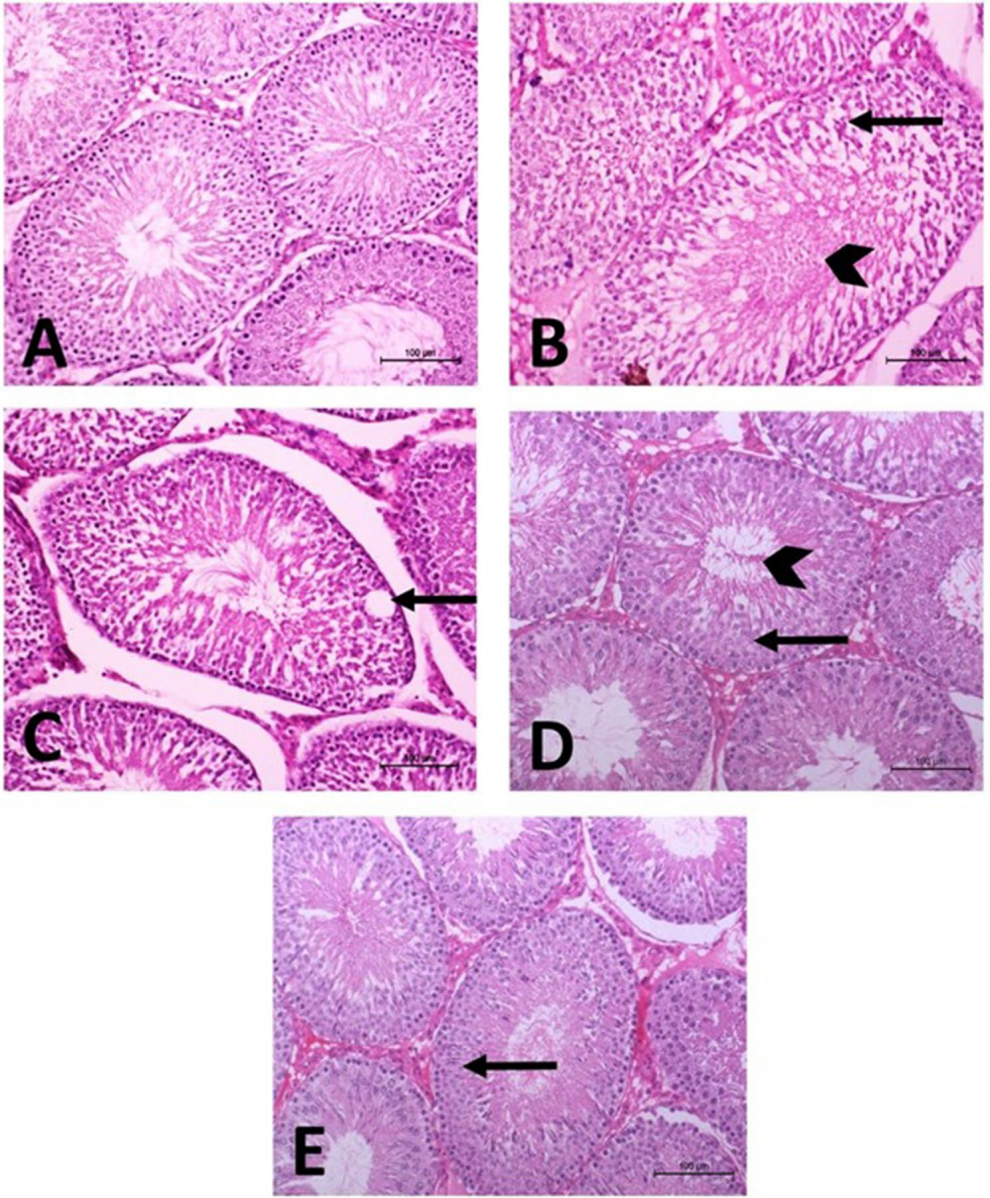
Effect of eltroxin and hesperidin on histopathological changes in the testis of CBZ-induced rats

H&E-stained sections of the testis from all experimental groups were represented in Fig. 7 and the detailed histopathological scores were summarized in Table 7. The control group revealed normal histology of seminiferous tubules which were oval or rounded with active spermatogenesis and lumen filled with frequent spermatozoa. Hypothyroid rats showed variable histopathological lesions in the form of severe irregular outlines of seminiferous tubules accompanied with degenerated germinal cells, deficient spermatogenesis, spermatogenic arrest, and vacuolization. Moderate degenerative spermatozoa in the affected lumens and necrosis of seminiferous tubules were also noticed. Severe empty seminiferous tubules could also be detected. In contrast, hypothyroid rats treated with ELT or HSP showed improvement in the testis sections with all mild pathological lesions. No influence of vacuoles in germ cells was observed in the hypothyroid rats treated with HSP.

**Table 7:** Grading of histopathological alterations obtained from testicular tissues of different experimental groups

Histopathological lesion	CON	CBZ	CBZ + ELT	CBZ + HSP
Irregular shape of STs	-	+++	+	+
Vacuoles in germ cells	-	+++	+	-
Degenerative changes in STs	-	+++	+	+
Tubular (germ cells) necrosis	-	++	+	+
No. of empty STs	-	+++	+	+
Degenerative spermatozoa	-	++	+	+

### Effect of ELT and HSP on histopathological changes in the kidney of CBZ-induced rats

**Fig. 8:**
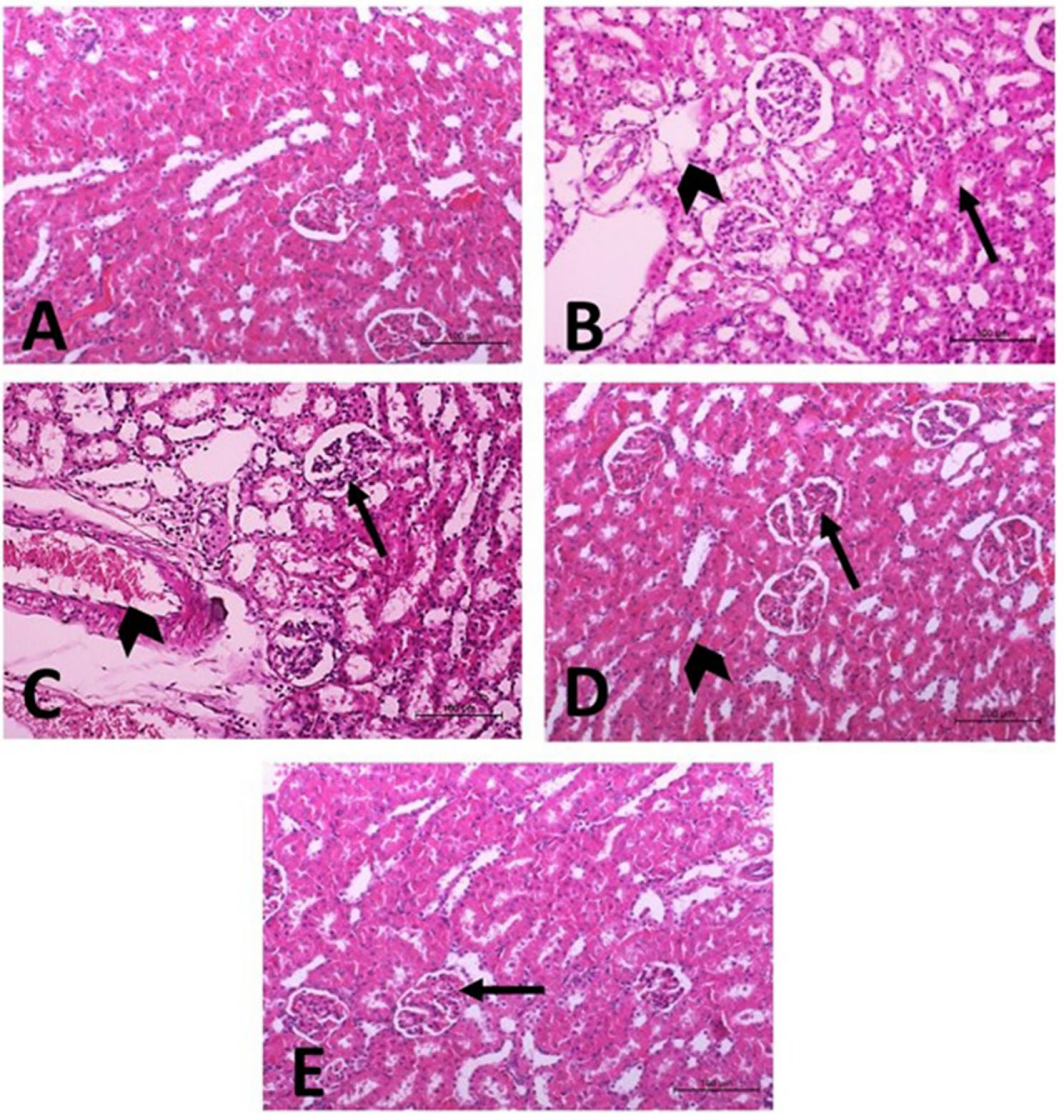
Effect of eltroxin and hesperidin on histopathological changes in the kidney of CBZ-induced rats

Figure 8 showed the photomicrographs of H&E stained kidney sections from all experimental groups by light microscopic examination, while the detailed histopathological scores were illustrated in Table 8. The control rats showed the normal structure of both glomeruli and renal tubules. Rats received CBZ showed multiple histopathological changes, including severe glomerular degeneration and tubular necrosis, moderate dilation of the Bowman capsular space, vacuolar degeneration of renal tubules, together with blood vessels congestion. Concomitant treatment of the hypothyroid rats with ELT showed glomeruli and renal tubules improvement with mild glomerular degeneration and tubular necrosis. Hypothyroid rats treated with HSP showed improvement in pathological lesions with mild degenerative changes of glomeruli.

**Table 8:** Grading of histopathological alterations obtained from renal tissues of different experimental groups

Histopathological lesion	CON	CBZ	CBZ + ELT	CBZ + HSP
Glomerulonephrosis	-	+++	+	+
Wide of Bowmans capsule	-	++	-	-
Tubulonephrosis	-	++	-	-
Necrosis of tubules	-	+++	+	-
Congestion	-	++	-	-

## Discussion

HPO is a common endocrine disorder caused by a decline in the THs released from the thyroid gland. In the present study, the induction of HPO was evidenced by the significantly decreased T3 and T4 and the significantly increased TSH relative to the control group. These outcomes were parallel with the result of Farag et al. [43]. The synthesis and release of TSH is negatively influenced by free T4 and T3 (unbounded to serum proteins) for keeping the levels of circulating THs within the normal range so when TH levels decrease, TSH secretion increases [44].

ELT or levothyroxine is the synthetic form of T4 hormone and is used to treat HPO. It is identical to the naturally produced thyroid hormone in human [45]. It has been proved in our work that ELT restored the changes in the levels of THs in harmony with the study of Abdel-Wahhab et al. [46]. ELT can increase T4 and T3 secretion by up-regulating TSH receptors [47], or by increasing serum protein level through the regulation of DNA transcription and protein synthesis; since the circulating T4 and T3 are protein bound hormones [48].

A significant modulation in the hormonal profile of the thyroid gland was also achieved in CBZ-induced rats after treatment with HSP. HSP has several positive pharmacological effects including anti-inflammatory, anticarcinogenic, antiviral, hypolipidemic, and hypoglycemic without any recorded negative side effects [49, 50]. Our present results were confirmed by Hegazy et al. [51] who reported that hypothyroid rats treated with HSP showed an ameliorative effect on THs levels. This positive effect could rely on the modulatory effects of flavanones on thyroid and pituitary functions [52], or the ameliorative effect of HSP on the histopathological and ultrastructural changes induced in the thyroid gland during the hypothyroidism state [53].

In accordance with Mohebbati et al. [54], the present findings demonstrated that CBZ-induced HPO is associated with nephrotoxicity as revealed by the increase in serum urea and creatinine levels. Urea and creatinine concentrations increase during kidney function failure [55]. On the contrary, co-treatment with ELT or HSP normalized urea and creatinine levels intensifying their renoprotective effect. According to Mooraki et al. [56], levothyroxine can reverse the decline in renal function caused by HPO. Additionally, in hypothyroid individuals with ischemic nephropathy, THs therapy quickly improved renal structure and function [57]. Hence, ELT effect may be exerted through the normalization of the THs levels. Similarly, it was previously recorded that HSP reduced the rise in serum urea and creatinine levels in rats [58] and mice [59] with nephrtotoxicity. The renoprotective effect of HSP may be explained based on its antioxidant property, enhancement of cellular antioxidant defenses and free radical scavenging ability [60].

In accordance with the results of Amra et al. [61], our study showed that testosterone, FSH and LH levels were decreased in hypothyroid rats. Furthermore, Mohamed and Bushra [62] suggested that the anti-thyroid drugs may reduce the liver’s capability to produce sex hormone-binding globulin (SHBG), which increases free androgen levels and inhibits the anterior pituitary’s ability to release gonadotropin-releasing hormone (GnRH), resulting in decreased FSH and LH levels. On the opposite hand, treatment with ELT or HSP showed a significant increase in testosterone, LH and FSH levels. The protective effect of HSP against testicular toxicity induced by drugs and chemicals was recorded earlier [63, 64]. The modulation of testicular hormonal levels may be accredited to the recovery of THs levels by ELT and HSP treatment. T3 could increase LH receptors and steroidogenesis of leydig cells [65]. T3 also stimulates testosterone secretion as a result of the activating action on leydig cells [66]. Similarly, T4 amplifies SHBG concentration, peripheral aromatization of androstenedione, and testosterone levels [67].

The HPO condition is associated with elevated pro-inflammatory cytokines as illustrated by high levels of TNF-α and IL-6 in renal and testicular tissues after CBZ induction. Raised pro-inflammatory marker levels have been reported in some previous investigations in patients with HPO [68, 69], and in animals′ renal tissue during a thyroid dysfunction state [70]. Gupta et al. [71] also indicated a significant increase in IL-6 levels in hypothyroid patients compared to controls and this increase was positively correlated with serum TSH levels. Conversely, a negative correlation was found between IL-6 and serum TT3 or TT4 levels [72].

Treatment with ELT significantly decreased IL-6 and TNF-α levels, demonstrating the importance of early treatment in the abrogation of inflammation. These findings were strengthened by Tayde et al. [73] and Goyal et al. [74] who reported a significant reduction in IL-6 and TNF-α levels in hypothyroid cases after treatment with levothyroxine. In contrary, Díez et al. [75] showed that increased TNF-α levels in hypothyroid patients did not return to normal following the restoration of thyroid function. Similarly, treatment of CBZ-induced rats with HSP markedly decreased renal and testicular IL-6 and TNF-α levels, indicating mitigated inflammation. Previous studies showed the anti-inflammatory effect of HSP in renal tissues [16, 76] and testicular tissues [77]. Under HPO conditions, HSP has been indicated to decrease inflammation in liver and lung as previously reported [51, 78].

THs can modulate oxidative metabolism and have a considerable impact on OS and the antioxidant system [79]. A positive correlation between lipid peroxidation and TSH was found; excess TSH directly produces OS [80]. Therefore, the oxidant/antioxidant status was necessarily investigated. Treating rats with CBZ in the current study showed a significant increase in lipid peroxidation level and a significant decrease in GSH content and the activities of the antioxidant enzymes (GST and GPX), indicating an OS in testis and kidney. Similar to our findings, several studies indicated that HPO induced OS in testis and kidney [81, 82]. CBZ can disturb the balance between ROS generation and the antioxidant defense systems [78]. This is consistent with Wang et al. [83] who believed that cell damage and testicular dysfunction observed during HPO resulted from the buildup of free radicals and increased lipid peroxidation and OS. Similarly, the kidney is susceptible to damage triggered by ROS because renal lipids contain a plenty of polyunsaturated fatty acids [84].

The present data showed that treatment with ELT and HSP reduced lipid peroxidation and improved enzymatic and non-enzymatic antioxidant defenses evidencing the ability of preventing oxidative stress as a mechanism to counteract HPO related renal and testicular damage. Chakrabarti et al. [85] showed that treatment of HPO with levothyroxine reduced the stress markers to a significant degree. THs have a critical role in minimizing oxidative stress by promoting the expression of antioxidant enzymes and acting as radical scavengers [86]. As well, HSP showed an antioxidant capacity in animal models of testicular [87, 88], hepato-renal [89, 90], and renal [12] toxicity.

To inspect the protective mechanism of ELT and HSP, we examined their effect on Nrf2/HO-1 signaling pathway. In the current work, hypothyroid rats showed a declined mRNA and protein expression of Nrf2 and HO-1, due to exaggerated generation of ROS. Several experiments demonstrated decreased Nrf2/ARE/HO-1 in situations with high ROS generation [91, 92]. Down-regulation in Nrf2/HO-1 pathway was illuminated not only in testicular toxicity [93], but also in nephrotoxicity [94, 95] caused by toxic agents.

On the opposite side, Nrf2 and HO-1 were significantly up-regulated in the hypothyroid group treated with HSP. Our results confirmed prior studies where HSP showed a cyto-protective effect by up-regulating Nrf2/HO-1 pathway in testicular toxicity [96] and nephrotoxicity [16, 97]. In the same trend, treating hypothyroid rats with ELT significantly increased Nrf2/HO-1 signaling. This result was confirmed by the increased expression of Nrf2 and HO-1 in the liver [51] and lung tissues [78] of hypothyroid rats treated with ELT.

Gene and protein expression of PPARγ was significantly decreased in both testis and kidney of hypothyroid rats. The inhibition of PPARγ expression was previously reported in testicular damage induced by toxic substances [98]. Inan et al. [99] also reported that testicular OS in rats can be increased following PPARγ inhibition. Additionally, there is a proof that PPARγ plays an anti-inflammatory effect in inflammatory disorders [100]. Increased expression of TNF-α and interleukin-1 beta (IL-1β) was suggested as the mechanism of PPARγ down-regulation in testicular tissues [101]. This ensures the negative correlation between PPARγ activation and TNF-α reduction that was documented in our study. Furthermore, increased OS and pro-inflammatory cytokines were reported to down-regulate PPARγ expression during renal failure [102].

Our findings reported that ELT or HSP exert beneficial effects on the testis and kidney through PPARγ activation. Several research studies reported that PPARγ agonists provided protection against HPO-induced toxicity in many organs [103–105]. Other reports suggested that PPARγ activation protected against renal failure models such as chronic renal allograft damage [106] and ischemia-reperfusion renal injury [107]. In addition, the antioxidant and anti-inflammatory properties of HSP against cyclophosphamide-induced hepatotoxicity [108] and isoproternol-induced cardiotoxicity [109] were performed through up-regulating PPARγ.

As a result, coactivation of Nrf2 and PPARγ appears to be the mediator of the antioxidant and anti-inflammatory effect of HSP and ELT. This can be explained since PPARγ response element is present in the Nrf2 gene’s promoter region [110]. Also, it has been shown that Nrf2 activates PPARγ and guards against oxidative damage [111].

Our histopathological findings were consistent with the biochemical results intensifying the results of Amra et al. [61] and Alhealy et al. [82] who clarified the testicular changes induced by HPO. Adverse effects in histological structure and semen parameters in adult male hypothyroid rats were also reported by Ibrahim et al. [112]. Co-administration of ELT or HSP showed improvement in testicular structure as previously reported [61, 87]. Their protective effect on HPO-induced testicular degeneration may be attributed to the antioxidant abilities, which eliminate free radicals to minimize lipid peroxidation and therefore preserve normal histological structure of testes [77, 113].

In the present study, hypothyroid rats showed histopathological changes in the renal tissues including damage in the glomeruli and tubules as were previously notified by Ubaid et al. [114]. Treatment of hypothyroid rats with ELT attenuated the severity of renal injuries except mild glomerular degeneration and tubular necrosis, while hypothyroid rats treated with HSP revealed improvement in renal tissues but some degenerative changes of glomeruli were still visible. The improvement in kidney structure provided further evidence of the antioxidant properties of ELT and HSP. Lashein et al. [115] reported that co-administration of levothyroxine with CBZ ameliorated renal histological changes. Similarly, Küçükler et al. [90] indicated that the antioxidant capacity of HSP was responsible for the protective efficacy against renal toxicity induced in rats by chlorpyrifos.

## Conclusions

This investigation proved that administration of ELT and HSP ameliorated THs concentration, testis hormonal profile, kidney function biomarkers, OS, and inflammation in rats with CBZ-induced HPO. These ameliorating effects were achieved through up-regulation of PPARγ and Nrf2/HO-1 signaling pathway. Histological changes of the testes and kidneys were also improved. Therefore, HSP could be a promising element in the development of a potent antihypothyroid agent. Also, we encourage patients with HPO to consume HSP rich foods along with medication to protect their body organs from OS and inflammation.

## Data Availability

The datasets analyzed during the current study are available from the corresponding author on a reasonable request.

## Abbreviations

ARE: Antioxidant response element
β-actin: Beta-actin
CBZ: Carbimazole
CMC: Carboxymethyl cellulose
ELT: Eltroxin
ELISA: Enzyme-linked immunosorbent assay
FSH: Follicle stimulating hormone
GSH: Reduced glutathione
GST: Glutathione-s-transferase
GPx: Glutathione peroxidase
GnRH: Gonadotropin-releasing hormone
HPO: Hypothyroidism
HSP: Hesperidin
HO-1: Heme oxygenase 1
H&E: Hematoxylin and eosin
IL-6: Interleukin 6
IL-1β: Interleukin-1 beta
LH: Luteinizing hormone
MDA: Malondialdehyde
Nrf2: Nuclear factor erythroid 2-related transcription factor 2
OS: Oxidative stress
PPARs: Peroxisome proliferator-activated receptors
PPAR-γ: Peroxisome proliferator-activated receptor-gamma
PPAR-α: Peroxisome proliferator-activated receptor-alpha
PPAR-β: Peroxisome proliferator-activated receptor-beta
PCR: Polymerase chain reaction
RAAS: Renin angiotensin aldosterone system
ROS: Reactive oxygen species
RXR: Retinoid X receptor
SOD: Superoxide dismutase
SHBG: Sex hormone-binding globulin
SD: Standard deviation
THs: Thyroid hormones
TT3: Total triiodothyronine
TT4: Total thyroxine
T3: 3,5,3’-Triiodothyronine
T4: Thyroxin or 3,5,3’,5’-tetraiodothyronine
TSH: Thyroid stimulating hormone (Thyrotropin)
TNF-α: Tumor necrosis factor alpha

## References

[1] Biondi B, Cooper DS (2019). Thyroid hormone therapy for hypothyroidism. Endocrine.

[2] Ross DS, Burch HB, Cooper DS, Carol Greenlee M, Laurberg P, Maia AL (2016). 2016 American Thyroid Association guidelines for diagnosis and management of hyperthyroidism and other causes of thyrotoxicosis. Thyroid.

[3] Chiovato L, Magri F, Carlé A (2019). Hypothyroidism in context: where we’ve been and where we’re going. Adv Ther.

[4] Baser H, Can U, Baser S, Yerlikaya FH, Aslan U, Hidayetoglu BT (2015). Assesment of oxidative status and its association with thyroid autoantibodies in patients with euthyroid autoimmune thyroiditis. Endocrine.

[5] Rhee CM (2016). The interaction between thyroid and kidney disease: an overview of the evidence. Curr Opin Endocrinol Diabetes Obes.

[6] La Vignera S, Vita R, Condorelli RA, Mongioì LM, Presti S, Benvenga S (2017). Impact of thyroid disease on testicular function. Endocrine.

[7] Hammes SR, Davis PJ (2015). Overlapping nongenomic and genomic actions of thyroid hormone and steroids. Best Pract Res Clin Endocrinol Metab.

[8] Ezz MK, Hassan RE, Esmat AY (2017). Pineapple juice supplementation activates thyroid gland and attenuates hyperlipidemia in rats. int j Bioscience.

[9] Sinha S, Chakraborty A, Mondal C, Chandra AK (2018). Effect of vitamin E acetate supplementation on thyroid hormone-sensitive organs following exogenous L-thyroxine treatment. Asian J Pharm Clin Res.

[10] Hughes SD, Ketheesan N, Haleagrahara N (2017). The therapeutic potential of plant flavonoids on rheumatoid arthritis. Crit Rev Food Sci Nutr.

[11] Hemanth Kumar B, Dinesh Kumar B, Diwan PV (2017). Hesperidin, a citrus flavonoid, protects against l-methionine-induced hyperhomocysteinemia by abrogation of oxidative stress, endothelial dysfunction and neurotoxicity in Wistar rats. Pharm Biol.

[12] Aditya G, Ajay S (2019). The pharmacological potential of hesperidin. IJBB.

[13] Aggarwal V, Tuli HS, Thakral F, Singhal P, Aggarwal D, Srivastava S (2020). Molecular mechanisms of action of hesperidin in cancer: recent trends and advancements. Exp Biol Med (Maywood).

[14] Chen M, Gu H, Ye Y, Lin B, Sun L, Deng W (2010). Protective effects of hesperidin against oxidative stress of tert-butyl hydroperoxide in human hepatocytes. Food Chem Toxicol.

[15] Sahu BD, Kuncha M, Sindhura GJ, Sistla R (2013). Hesperidin attenuates cisplatin-induced acute renal injury by decreasing oxidative stress, inflammation and DNA damage. Phytomedicine.

[16] Subramanian P, Anandan R, Jayapalan JJ, Hashim OH (2015). Hesperidin protects gentamicin-induced nephrotoxicity via Nrf2/HO-1 signaling and inhibits inflammation mediated by NF-κB in rats. J Funct Foods.

[17] Li Y, Kandhare AD, Mukherjee AA, Bodhankar SL (2019). Acute and sub-chronic oral toxicity studies of hesperidin isolated from orange peel extract in Sprague Dawley rats. Regul Toxicol Pharmacol.

[18] Renaud CO, Ziros PG, Chartoumpekis DV, Bongiovanni M, Sykiotis GP (2019). Keap1/Nrf2 signaling: a new player in thyroid pathophysiology and thyroid cancer. Front Endocrinol (Lausanne).

[19] Ziros PG, Habeos IG, Chartoumpekis DV, Ntalampyra E, Somm E, Renaud CO (2018). NFE2-related transcription factor 2 coordinates antioxidant defense with thyroglobulin production and iodination in the thyroid gland. Thyroid.

[20] Lee JM, Li J, Johnson DA, Stein TD, Kraft AD, Calkins MJ (2005). Nrf2, a multi-organ protector?. FASEB J.

[21] Barish GD, Narkar VA, Evans RM (2006). PPARδ: a dagger in the heart of the metabolic syndrome. J Clin Investig.

[22] Michalik L, Wahli W (2008). PPARs mediate lipid signaling in inflammation and cancer. PPAR Res.

[23] Olia Bagheri F, Alizadeh A, Sadighi Gilani MA, Shahhoseini M (2021). Role of peroxisome proliferator-activated receptor gamma (PPARγ) in the regulation of fatty acid metabolism related gene expressions in testis of men with impaired spermatogenesis. Reprod Biol.

[24] Turturro F, Oliver R, Friday E, Nissim I, Welbourne T (2007). Troglitazone and pioglitazone interactions via PPAR-γ-independent and-dependent pathways in regulating physiological responses in renal tubule-derived cell lines. Am J Physiol Cell Physiol.

[25] Mahmoud AM, Germoush MO, Alotaibi MF, Hussein OE (2017). Possible involvement of Nrf2 and PPARγ up-regulation in the protective effect of umbelliferone against cyclophosphamide-induced hepatotoxicity. Biomed Pharmacother.

[26] Mahmoud AM, Hussein OE, Hozayen WG, Abd el-Twab SM (2017). Methotrexate hepatotoxicity is associated with oxidative stress, and down-regulation of PPARγ and Nrf2: protective effect of 18β-Glycyrrhetinic acid. Chem Biol Interact.

[27] Abdel Reheim ES, El-Twab A (2014). Effect of carrot oil supplementation on oxidative stress in experimentally-induced hypothyroidism and hyperthyroidism in albino rats. Egypt J Exp Biol (Zoo).

[28] Paget G, Barnes J (1964). Interspecies dosage conversion scheme in evaluation of results and quantitative application in different species. Evaluation drug Activities: Pharmacometrics.

[29] Estruel-Amades S, Massot-Cladera M, Pérez-Cano FJ, Franch À, Castell M, Camps-Bossacoma M (2019). Hesperidin effects on gut microbiota and gut-associated lymphoid tissue in healthy rats. Nutrients.

[30] Gage GJ, Kipke DR, Shain W. Whole animal perfusion fixation for rodents. J Vis Exp. 2012:65):3564. 10.3791/3564 PMC3476408 22871843

[31] Thakur C, Saikia TC, Yadav N (1997). Total serum levels of triiodothyronine (T3) thyroxine (T4) and thyrotropine (TSH) in school going children of dibrugarh district: an endemic goitre region of assam. Indian J Physiol Pharmacol.

[32] Maes M, Mommen K, Hendrickx D, Peeters D, D’Hondt P, Ranjan R (1997). Components of biological variation, including seasonality, in blood concentrations of TSH, TT3, FT4, PRL, cortisol and testosterone in healthy volunteers. Clin Endocrinol (Oxf).

[33] Burger HG, Patel YC (1977). Thyrotrophin releasing hormone—TSH. Clin Endocrinol Metab.

[34] Coulombe J, Favreau L (1963). A new simple semimicro method for colorimetric determination of urea. Clin Chem.

[35] Larsen K (1972). Creatinine assay in the presence of protein with LKB 8600 reaction rate Analyser. Clin Chim Acta.

[36] Preuss HG, Jarrell ST, Scheckenbach R, Lieberman S, Anderson RA (1998). Comparative effects of chromium, vanadium and Gymnema sylvestre on sugar-induced blood pressure elevations in SHR. J Am Coll Nutr.

[37] Beutler E, Duron O, Kelly BM (1963). Improved method for the determination of blood glutathione. J lab clin med.

[38] Matkovics B, Szabo L, Varga IS (1998). Determination of enzyme activities in lipid peroxidation and glutathione pathways (in Hungarian). Lab Diagn.

[39] Mannervik B, Guthenberg C (1981). Glutathione transferase (human placenta). Methods Enzymol.

[40] Bancroft JD, Gamble M (2008). Theory and practice of histological techniques.

[41] Abdel-Raheem IT, Abdel-Ghany AA, Mohamed GA (2009). Protective effect of quercetin against gentamicin-induced nephrotoxicity in rats. Biol Pharm Bull.

[42] Livak KJ, Schmittgen TD (2001). Analysis of relative gene expression data using real-time quantitative PCR and the 2 – ∆∆CT method. Methods.

[43] Farag EA, Filobbos SA, Afifi NM, Mahmoud ST, Alghandour SM (2023). Thyroxine restores hippocampal neurogenesis and synaptogenesis in a male rat model of carbimazole-induced hypothyroidism: a histological study. Beni-Suef univ j Basic appl sci.

[44] Uduak OA, Ani EJ, Etoh ECI, Macstephen AO (2014). Comparative effect of Citrus sinensis and carbimazole on serum T4, T3 and TSH levels. Niger Med J.

[45] Ochani S, Siddiqui A, Adnan A (2022). Adverse effects of long-term levothyroxine therapy in subclinical hypothyroidism. Ann Med Surg.

[46] Abdel-Wahhab KG, Mourad HH, Mannaa FA, Morsy FA, Hassan LK, Taher RF (2019). Role of ashwagandha methanolic extract in the regulation of thyroid profile in hypothyroidism modeled rats. Mol Biol Rep.

[47] Vaidya B, Pearce SH (2008). Management of hypothyroidism in adults. BMJ.

[48] Laurberg P (1984). Mechanisms governing the relative proportions of thyroxine and 3, 5, 3′-triiodothyronine in thyroid secretion. Metabolism.

[49] Tabeshpour J, Hosseinzadeh H, Hashemzaei M, Karimi G (2020). A review of the hepatoprotective effects of hesperidin, a flavanon glycoside in citrus fruits, against natural and chemical toxicities. Daru.

[50] Tao G, Dagher F, Moballegh A, Ghose R (2020). Role of oxidative stress in the efficacy and toxicity of herbal supplements. Curr Opin Toxicol.

[51] Hegazy W, Abdel-Moneim A, Abdel-Rehiem ES, Salah M, Abdul-Hamid M (2022). The protective effect of hesperidin on the liver of hypothyroid rats mediated by nuclear factor erythroid 2-related factor 2-dependent activation of heme oxygenase 1. J Mol Histol.

[52] Miler M, Živanović J, Ajdžanović V, Milenkovic D, Jarić I, Šošić-Jurjević B (2020). Citrus flavanones upregulate thyrotroph Sirt1 and differently affect thyroid Nrf2 expressions in old-aged Wistar rats. J Agric Food Chem.

[53] Hegazy W, Abdul-Hamid M, Abdel-Rehiem ES, Abdel-Moneim A, Salah M (2023). The protective impact of hesperidin against carbimazole-induced hypothyroidism, via enhancement of inflammatory cytokines, histopathological alterations, and Nrf2/HO-1. Environ Sci Pollut Res Int.

[54] Mohebbati R, Hosseini M, Haghshenas M, Nazariborun A, Beheshti F (2017). Th e eff ects of Nigella sativa extract on renal tissue oxidative damage during neonatal and juvenile growth in propylthiouracil-induced hypothyroid rats. Endocr Regul.

[55] Higgins C. Urea and creatinine concentration, the urea: creatinine ratio. Acute Care Test Hand. 2016:1–8.

[56] Mooraki A, Broumand B, Neekdoost F, Amirmokri P, Bastani B (2003). Reversible acute renal failure associated with hypothyroidism: report of four cases with a brief review of literature. Nephrol (Carlton).

[57] Makino Y, Fujii T, Kuroda S, Inenaga T, Kawano Y, Takishita S (2000). Exacerbation of renal failure due to hypothyroidism in a patient with ischemic nephropathy. Nephron.

[58] Moustafa S, Hashish R, Abdel-Karim R (2022). The possible ameliorative effect of hesperidin administration in aluminum phosphide induced acute nephrotoxicity in adult albino rats. Ain-Shams J Forensic Med Clin Toxicol.

[59] Gelen V, Şengül E, Yıldırım S, Senturk E, Tekin S, Kükürt A (2021). The protective effects of hesperidin and curcumin on 5-fluorouracil–induced nephrotoxicity in mice. Environ Sci Pollut Res Int.

[60] Parhiz H, Roohbakhsh A, Soltani F, Rezaee R, Iranshahi M (2015). Antioxidant and anti-inflammatory properties of the citrus flavonoids hesperidin and hesperetin: an updated review of their molecular mechanisms and experimental models. Phytother Res.

[61] Amra EA, El Rehim SAA, Lashein FM, Shoaeb HS (2022). Effect of a bradykinin potentiating factor separated from honey bee venom on thyroid gland and testis in hypothyroid white rats. j Basic appl zool.

[62] Mohamed HZ, Bushra RR (2017). Effects of simultaneous melatonin administration on the testis of the experimentally induced hyper-and hypothyroidism in the adult male albino rat. Egypt J Histol.

[63] Khamis T, Hegazy AA, El-Fatah SSA, Abdelfattah ER, Abdelfattah MMM, Fericean LM (2023). Hesperidin mitigates Cyclophosphamide-Induced Testicular Dysfunction via altering the hypothalamic pituitary gonadal Axis and testicular steroidogenesis, inflammation, and apoptosis in male rats. Pharmaceuticals.

[64] Kasem SE, Abdelnaby AA, Mohammed PA, Hemdan SB, El-Fattah A, Rasha M (2022). Protective effect of Hesperidin on kidneys and testes of adult male rats exposed to Bisphenol A. Egypt J Hosp Med.

[65] Manna PR, Jo Y, Stocco DM (2007). Regulation of Leydig cell steroidogenesis by extracellular signal-regulated kinase 1/2: role of protein kinase A and protein kinase C signaling. J Endocrinol.

[66] Maran RRM, Arunakaran J, Aruldhas MM (2000). T3 directly stimulates basal and modulates LH induced testosterone and oestradiol production by rat leydig cells in vitro. Endocr J.

[67] Longcope C, Feldman HA, Mckinlay JB, Araujo AB (2000). Diet and sex hormone-binding globulin. J Clin Endocrinol Metab.

[68] Taddei S, Caraccio N, Virdis A, Dardano A, Versari D, Ghiadoni L (2006). Low-grade systemic inflammation causes endothelial dysfunction in patients with Hashimoto’s thyroiditis. J Clin Endocrinol Metab.

[69] Türemen EE, Çetinarslan B, Sahin T, Cantürk Z, Tarkun I (2011). Endothelial dysfunction and low grade chronic inflammation in subclinical hypothyroidism due to autoimmune thyroiditis. Endocr J.

[70] Ajayi A, Akhigbe R, Ajayi L (2018). Influence of thyroid dysfunction on Urea/Creatinine ratio: possible role of TNF-α and IL-6. Intjmedbiomedres.

[71] Gupta G, Sharma P, Kumar P, Itagappa M (2015). Study on subclinical hypothyroidism and its association with various inflammatory markers. J Clin Diagn Res.

[72] Davies PH, Black EG, Sheppard MC, Franklyn JA (1996). Relation between serum interleukin-6 and thyroid hormone concentrations in 270 hospital in‐patients with non‐thyroidal illness. Clin Endocrinol (Oxf).

[73] Tayde PS, Bhagwat NM, Sharma P, Sharma B, Dalwadi PP, Sonawane A (2017). Hypothyroidism and depression: are cytokines the link?. Indian J Endocrinol Metab.

[74] Goyal S, Dixit A, Vaney N, Madhu S. Serum levels of inflammatory markers in newly diagnosed hypothyroid patients before and after levothyroxine therapy. J Clin Diagn Res. 2022;16(9).

[75] Díez JJ, Hernanz A, Medina S, Bayón C, Iglesias P (2002). Serum concentrations of tumour necrosis factor-alpha (TNF‐α) and soluble TNF‐α receptor p55 in patients with hypothyroidism and hyperthyroidism before and after normalization of thyroid function. Clin Endocrinol (Oxf).

[76] Turk E, Kandemir FM, Yildirim S, Caglayan C, Kucukler S, Kuzu M (2019). Protective effect of hesperidin on sodium arsenite-induced nephrotoxicity and hepatotoxicity in rats. Biol Trace Elem Res.

[77] Tekin S, Çelebi F (2022). Investigation of the effect of hesperidin on some reproductive parameters in testicular toxicity induced by B isphenol A. Andrologia.

[78] Hegazy W, Sakr HI, Abdul Hamid M, Abdelaziz MA, Salah M, Abdel Rehiem ES (2023). Hesperidin attenuates Hypothyroidism-Induced Lung damage in adult albino rats by modulating oxidative stress, nuclear factor Kappa-B pathway, proliferating Cell Nuclear Antigen and Inflammatory cytokines. Biomedicines.

[79] Chainy GB, Sahoo DK (2020). Hormones and oxidative stress: an overview. Free Radic Res.

[80] Haribabu A, Reddy VS, Pallavi C, Bitla AR, Sachan A, Pullaiah P (2013). Evaluation of protein oxidation and its association with lipid peroxidation and thyrotropin levels in overt and subclinical hypothyroidism. Endocrine.

[81] Ayuob N, Balgoon MJ, El-Mansy AA, Mubarak WA, Firgany AE-DL (2020). Thymoquinone Upregulates Catalase Gene expression and preserves the structure of the renal cortex of Propylthiouracil-Induced hypothyroid rats. Oxid Med Cell Longev.

[82] Alhealy E, Alqazzaz M, AL-Nuaimy WM (2021). Impact of Selenium on Structural Changes Induced by Hypothyroidism in Adult Male Rat׳ s Testis. Iraqi J Pharm.

[83] Wang J-L, Zhang H-J, Wang H-L, Wang J-W, Gou P-H, Ye Z-H (2015). Influence of hypothyroidism on oxidative stress, c-Fos expression, cell cycle, and apoptosis in rats testes. Toxicol Environ Chem.

[84] Ozbek E (2012). Induction of oxidative stress in kidney. Int J Nephrol.

[85] Chakrabarti SK, Ghosh S, Banerjee S, Mukherjee S, Chowdhury S (2016). Oxidative stress in hypothyroid patients and the role of antioxidant supplementation. Indian J Endocrinol Metab.

[86] Villanueva I, Alva-Sánchez C, Pacheco-Rosado J (2013). The role of thyroid hormones as inductors of oxidative stress and neurodegeneration. Oxid Med Cell Longev.

[87] Afolabi OK, Wusu AD, Ugbaja R, Fatoki JO. Aluminium phosphide-induced testicular toxicity through oxidative stress in Wistar rats: ameliorative role of hesperidin. Toxicol Res Appl. 2018;2.

[88] Ileriturk M, Kandemir O, Akaras N, Simsek H, Genc A, Kandemir FM (2023). Hesperidin has a protective effect on paclitaxel-induced testicular toxicity through regulating oxidative stress, apoptosis, inflammation and endoplasmic reticulum stress. Reprod Toxicol.

[89] Abdelaziz RM, Abdelazem AZ, Hashem KS, Attia YA (2020). Protective effects of hesperidin against MTX-induced hepatotoxicity in male albino rats. Naunyn Schmiedebergs Arch Pharmacol.

[90] Küçükler S, Çomaklı S, Özdemir S, Çağlayan C, Kandemir FM (2021). Hesperidin protects against the chlorpyrifos-induced chronic hepato‐renal toxicity in rats associated with oxidative stress, inflammation, apoptosis, autophagy, and up‐regulation of PARP‐1/VEGF. Environ Toxicol.

[91] Mohammed ET, Hashem KS, Ahmed AE, Aly MT, Aleya L, Abdel-Daim MM (2020). Ginger extract ameliorates bisphenol A (BPA)-induced disruption in thyroid hormones synthesis and metabolism: involvement of Nrf-2/HO-1 pathway. Sci Total Environ.

[92] Mahmoud AM, Hozayen WG, Ramadan SM (2017). Berberine ameliorates methotrexate-induced liver injury by activating Nrf2/HO-1 pathway and PPARγ, and suppressing oxidative stress and apoptosis in rats. Biomed Pharmacother.

[93] Abdel-Wahab BA, Alkahtani SA, Elagab EA (2020). Tadalafil alleviates cisplatin-induced reproductive toxicity through the activation of the Nrf2/HO-1 pathway and the inhibition of oxidative stress and apoptosis in male rats. Reprod Toxicol.

[94] Albarakati AJA, Baty RS, Aljoudi AM, Habotta OA, Elmahallawy EK, Kassab RB (2020). Luteolin protects against lead acetate-induced nephrotoxicity through antioxidant, anti-inflammatory, anti-apoptotic, and Nrf2/HO-1 signaling pathways. Mol Biol Rep.

[95] Abdou K, Moselhy WA, Mohamed HM, El-Nahass E-S, Khalifa AG (2019). Moringa oleifera leaves extract protects titanium dioxide nanoparticles-induced nephrotoxicity via Nrf2/HO-1 signaling and amelioration of oxidative stress. Biol Trace Elem Res.

[96] Gur C, Kandemir O, Kandemir FM (2022). Investigation of the effects of hesperidin administration on abamectin-induced testicular toxicity in rats through oxidative stress, endoplasmic reticulum stress, inflammation, apoptosis, autophagy, and JAK2/STAT3 pathways. Environ Toxicol.

[97] Morsy MA, El-Sheikh AAK, Ibrahim ARN, El-Daly M (2021). Protection of Hesperidin against Methotrexate-Induced Nephrotoxicity may be mediated by Nrf2/HO-1 pathway. Indian J Pharm Educ Res.

[98] Abu-Risha SE, Mousa MA, Elsisi AE (2022). Protective role of irbesartan against cyclophosphamide-induced testicular damage in rats via up-regulating PPAR-γ signaling and ameliorating NF-κB/NLRP3/IL-18 inflammatory axis. Life Sci.

[99] Inan M, Basaran U, Dokmeci D, Kanter M, Yalcin O, Aydogdu N (2007). Rosiglitazone, an agonist of peroxisome proliferator-activated receptor‐gamma, prevents contralateral testicular ischaemia–reperfusion injury in prepubertal rats. Clin Exp Pharmacol Physiol.

[100] Martin H (2009). Role of PPAR-gamma in inflammation. Prospects for therapeutic intervention by food components. Mutat Res.

[101] Ghobadi E, Moloudizargari M, Asghari MH, Abdollahi M (2017). The mechanisms of cyclophosphamide-induced testicular toxicity and the protective agents. Expert Opin Drug Metab Toxicol.

[102] Itoh H, Doi K, Tanaka T, Fukunaga Y, Hosoda K, Inoue G (1999). Hypertension and insulin resistance: role of peroxisome proliferator-activated receptor γ. Clin Exp Pharmacol Physiol.

[103] Baghcheghi Y, Salmani H, Beheshti F, Shafei MN, Sadeghnia HR, Soukhtanloo M (2019). Effects of PPAR-γ agonist, pioglitazone on brain tissues oxidative damage and learning and memory impairment in juvenile hypothyroid rats. Int J Neurosci.

[104] Baghcheghi Y, Beheshti F, Hosseini M, Gowhari-Shabgah A, Ali-Hassanzadeh M, Hedayati-Moghadam M (2022). Cardiovascular protective effects of PPARγ agonists in hypothyroid rats: protection against oxidative stress. Clin Exp Hypertens.

[105] Baghcheghi Y, Beheshti F, Salmani H, Soukhtanloo M, Hosseini M (2016). Protective effect of PPARγ agonists on cerebellar tissues oxidative damage in hypothyroid rats. Neurol Res Int.

[106] Kiss E, Popovic ZV, Bedke J, Adams J, Bonrouhi M, Babelova A (2010). Peroxisome proliferator-activated receptor (PPAR) γ can inhibit chronic renal allograft damage. Am J Pathol.

[107] Doi S, Masaki T, Arakawa T, Takahashi S, Kawai T, Nakashima A (2007). Protective effects of peroxisome proliferator-activated receptor γ ligand on apoptosis and hepatocyte growth factor induction in renal ischemia-reperfusion injury. Transplantation.

[108] Mahmoud AM (2014). Hesperidin protects against cyclophosphamide-induced hepatotoxicity by upregulation of PPARγ and abrogation of oxidative stress and inflammation. Can J Physiol Pharmacol.

[109] Agrawal YO, Sharma PK, Shrivastava B, Arya DS, Goyal SN (2014). Hesperidin blunts streptozotocin-isoproternol induced myocardial toxicity in rats by altering of PPAR-γ receptor. Chem Biol Interact.

[110] Shih AY, Imbeault S, Barakauskas V, Erb H, Jiang L, Li P (2005). Induction of the Nrf2-driven antioxidant response confers neuroprotection during mitochondrial stress in vivo. J Biol Chem.

[111] Cho H-Y, Gladwell W, Wang X, Chorley B, Bell D, Reddy SP (2010). Nrf2-regulated PPARγ expression is critical to protection against acute lung injury in mice. Am J Respir Crit Care Med.

[112] Ibrahim A, Mohammed N, Eid K, Abomughaid M, Abdelazim A, Aboregela A, Hypothyroidism (2021). Morphological and metabolic changes in the testis of adult albino rat and the amelioration by alpha-lipoic acid. Folia Morphol.

[113] Masullo LF, Magalhães RA, Lemes RPG, de Almeida Filho TP, De Castro MF, Maia Filho PA (2018). Levothyroxine replacement improves oxidative status in primary hypothyroidism. Front Endocrinol (Lausanne).

[114] Ubaid MM, Kadhim SH, Musa AU, Abed al-kareem Z, Aziz ND (2018). The analysis of the protective feature of Nigella sativa in reducing carbimazole toxicity including liver and kidney parameters on albino male rats. Sci J Med Res.

[115] Lashein FEDM, Abd El Rehim SA, Abu Amra E (2022). Ameliorative effects of BPF separated from Honey Bee venoms on liver and kidney functions in Hypothyroidic Male Rat’s model. Sohag J Sci.

